# MET alterations in advanced pulmonary sarcomatoid carcinoma

**DOI:** 10.3389/fonc.2022.1017026

**Published:** 2022-09-23

**Authors:** Chen Gong, Huihua Xiong, Kai Qin, Jianhua Wang, Yi Cheng, Jing Zhao, Jing Zhang

**Affiliations:** Department of Oncology, Tongji Hospital, Tongji Medical College, Huazhong University of Science and Technology, Wuhan, China

**Keywords:** MET, pulmonary sarcomatoid carcinoma, skipping mutation, amplification, overexpression

## Abstract

Pulmonary sarcomatoid carcinoma (PSC) is a rare subset of NSCLC that accounts for about 0.5-1% of all primary lung carcinoma, and its malignant biological behavior is more aggressive than other pathological types of lung cancer. Recent studies have reported a variety of gene mutations associated with the occurrence, development and treatment of PSC, especially the mesenchymal-epithelial transition (*MET*) proto-oncogene alterations, including the exon 14 (*MET*ex14) skipping mutations as well as the amplification and overexpression of *MET* gene, which are associated with molecularly targeted therapy for PSC. *MET*ex14 skipping mutation is the most common and well-studied mutation type, occurring in about 22-31.8% of PSC patients, while the prevalence of *MET* amplification is reported as 4.8-13.6% and *MET* ovexpression is about 20.2%. Molecular pathology tests, including IHC and NGS, are valuable in determining the prognosis of patients with PSC and helping to determine the treatment. The existing clinical data have confirmed the efficacy of *MET*-TKI in PSC patients with *MET* alteration, among which the clinical study of Savolitinib has enrolled the largest proportion of PSC patients and achieved relatively good efficacy, but more clinical researches are still needed. The multi-disciplinary team may maximize the optimal treatment options for patients with the advanced PSC.

## Introduction

Non-small cell lung cancer (NSCLC) is the most common type of lung cancer, accounting for approximately 85% of all lung cancer with significant heterogeneity and it may be associated with some known and/or unknown driver gene changes (1). According to the Non-Small Cell Lung Cancer, Version 3.2022, NCCN Clinical Practice Guidelines in Oncology, NSCLC can be further classified into adenocarcinoma, squamous carcinoma, adenosquamous carcinoma, large cell carcinoma, and sarcomatoid carcinoma (2). Twenty years ago, there were limited treatments, such as surgery, radiotherapy and chemotherapy for advanced NSCLC, and the overall prognosis was dismal (3). However, the prognosis of advanced NSCLC has improved dramatically. And one of the important reasons is the discovery of the driver genes, their somatic genomic alterations (also known as molecular biomarkers) includes gene mutations and fusions, and the emergence of molecular targeted drugs (2–4). In the NCCN guidelines, the recommended genes for genetic testing in most patients with advanced NSCLC include *ALK*, *BRAF*, *EGFR*, *KRAS*, *MET*, *NTRK1/2/3*, *RET* and *ROS1*. The broad genomic testing can be used to assess mechanisms of drug resistance in patients who have relapsed after the targeted therapies, to distinguish primary lung cancer from intrapulmonary metastasis, and to help determining suitability for certain molecular-driven clinical trials (2).

Pulmonary sarcomatoid carcinoma (PSC) is a rare subset of NSCLC that accounts for about 0.5-1% of all primary lung carcinoma, and its malignant biological behavior is more aggressive than other pathological types of lung cancer (5–7). According to the World Health Organization (WHO) Classification of Lung Tumors (2015 version), PSC is defined as a group of poorly differentiated NSCLC containing a component of sarcoma-like elements or true sarcomatous areas that is currently solely based on morphological characteristics, and is divided into five subtypes such as spindle cell carcinoma (a carcinoma almost completely composed of epithelial spindle cells), giant cell carcinoma (a carcinoma almost entirely composed of tumor giant cells), pleomorphic carcinoma (a poorly differentiated NSCLC that contains at least 10% spindle and/or giant cells or a carcinoma consisting only of spindle and giant cells), carcinosarcoma (a mix of NSCLC and true sarcoma) and biphasic pulmonary blastoma (a tumor composed of embryonal-type epithelial elements and primitive mesenchymal stroma) (6, 8).

PSC usually occurs in older men with a history of smoking, and its clinical symptoms are non-specific compared with other types of NSCLC, such as cough, chest pain, hemoptysis, and dyspnea, which makes early detection and diagnosis difficult to some extent (5, 6). PSC has a very poor prognosis, with overall survival (OS) and 5-year survival rate significantly lower than other types of lung cancer. In different studies, 5-year survival for sarcomatoid carcinoma has been reported ranging from 20.1% to 36.1%, with less than 5% survival and less than 7 months survival in advanced stage (5, 6). Due to the rapid progression of PSC and its insensitivity to conventional chemotherapy agents, previously, many patients with PSC do not have the opportunity to receive a second-line chemotherapy or to try other novel antitumor agents.

Although the precise molecular characterization of PSC is largely unexplored, many studies have found that a variety of gene mutations may be associated with the occurrence, development, and treatment of PSC. Several recent studies have confirmed the mesenchymal-epithelial transition (*MET*) proto-oncogene alterations in PSC, including *MET* exon 14 (*MET*ex14) skipping mutations and *MET* amplification, with an incidence of approximately 22% and association with molecular-targeted therapy for PSC (6). This review aims to update the molecular pathology and clinical features of *MET* gene alteration in PSC.

## Wild type *MET* structure and function

The *MET* gene is located on the long arm of human chromosome 7q21-q31 (125kb long) and contains 21 exons. The *MET* proto-oncogene encodes a membrane *MET* tyrosine kinase receptor that mainly expressed in epithelial cells, also known as hepatocyte growth factor (HGF) receptors, which have been identified as a tumor driver gene and potential target of NSCLC (9). *MET* can bind with HGF with high affinity and induce a series of biological effects, and it is frequently associated with and functionally supports the Epithelial-to-Mesenchymal Transition, which is mainly manifested to stimulate cell proliferation, survival, invasion and migration in tumors (8). In other words, the ectopic activation of *MET* pathways can drive the development, growth, and metastasis of various malignancies, including lung cancer, breast cancer, cervical cancer, gastric cancer and colon cancer (10).

Being structured with a 50kD α chain and a 145kD β chain linked by disulfide bonds, MET protein also contain an N-terminal extracellular binding domain, a transmembrane helical domain, and an intracellular C-terminal domain with tyrosine kinase activity (11). The extracellular domain contains three distinct functional regions, including the Semaphorin (SEMA) domain covering the whole α chain and the N-terminal of some β chain, cystine-rich MET-related domain (Plexins-semaphorins-Integrins, PSI) containing four disulphide bonds and four immunoglobulin-plexin transcription regions (IPT) (3). The intracellular domain (957-1390 amino acid residues) after the transmembrane helical domain also consists of three regulatory regions, including the juxtamembrane (JM) domain containing Tyr1003 and Ser985 phosphorylation sites, the catalytic domain containing Y1234 and Y1235 phosphorylation sites, and the C-terminal multifunctional binding region containing Y1349 and Y1356 acting as a docking site for adaptor proteins, which leads to downstream signaling *via* phosphoinositide 3-kinase (PI3K)/AKT (protein kinase B), signal transducer and activator of transcription protegins (STAT), mitogen-activated protein kinase (MAPK), Wnt/beta-catenin, extracellular signal regulated kinase (ERK), mammalian target of rapamycin (mTOR)and nuclear factor-kB (NF-kB) (3, 11, 12).

HGF is the only known natural ligand of MET, and its binding to MET leads to receptor dimerization and phosphorylation of Y1234 and Y1235 tyrosine residues in the kinase domain’s catalytic loop and autophosphorylation of the carboxy-terminal bidentate substrate-binding sites 1349 and 1356, which can activates RTK-mediated downstream signaling pathways mentioned above (3, 11). These signaling transduction pathways are widely involved in cell proliferation, survival, cell motility, embryogenesis, organogenesis, angiogenesis, epithelial-mesenchymal transition and many other important biological behaviors in normal cells (13).

MET dysfunction is considered to be one of the driver event of lung cancer, which is often caused by gene copy number amplification, receptor protein overexpression, genetic sequence variations; exon 14 JM skipping mutations cause alternative splicing variant, and *MET* gene fusions (14). Many studies have reported that the frequency of *MET* alteration in PSC is higher than that in other types of lung cancer. At present, the most important *MET* alteration in the field of PCS is *MET* overexpression, *MET* amplification, and *MET*ex14 skipping mutations. At the same time, routine application of genetic testing in clinical practice can identify potential genetic biomarkers for developing targeted treatments and provide treatment options in addition to surgery, radiation, and chemotherapy.

## *MET* mutations

*MET* mutations can be detected in 3% to 5% of patients with non-small cell lung cancer (mainly adenocarcinoma) and occur more frequently in PSC, and *MET*ex14 skipping mutation is the most common and well-studied mutation type, occurring in about 3%-4% of adenocarcinoma patients and 22% of PSC patients (15, 16). Therefore, we will focus on the *MET*14 exon skipping mutation, including the biology, genetic testing, diagnosis and related molecular targeted therapy strategies of *MET*ex14 skipping mutation in this section.

The molecular mechanism of *MET*ex14 skipping mutation in NSCLCs was reported by Kong Beltran et al. in 2006 (17). Exon 14 encodes the 47-amino acid JM domain of the *MET* receptor, a key regulatory region that prevents MET hyperactivation. When MET mutation occurs, the binding sites for Y1003 and c-CBL are lost and the process of CBL-mediated MET protein degradation is impaired, MET receptors are aggregated, and MET oncogenic signals are overactivated (18). *MET*ex14 skipping mutation is considered to be an independent lung cancer driver, which is usually mutually exclusive with other lung cancer driver genes such as *EGFR*, *ALK* and *ROS1*, and is also associated with poor prognosis of lung cancer, including sarcomatoid cancer (19). Therefore, some studies recognized the lung cancer with *MET*ex14 skipping mutation as an independent molecular subtype and carried out individualized treatment (20).

Compared with the common type of non-small cell lung cancer, the incidence of *MET*ex14 skipping mutation is significantly higher in PSC (7). It is important to improve the sensitivity and specificity of the genetic testing for *MET*ex14 skipping mutation, which is an independent driver mutation of NSCLC without exception for PSC and its clinical significance is more prominent. Reverse transcriptase polymerase chain reaction(RT-PCR) and Sanger sequencing have both been used to detect *MET* mutations, but these targeted methods are rarely used because of the efficiency of detection (21). Compared with testing other lung cancer driver genes, such as *EGFR* and *KRAS*, the application of NGS in the diagnosis of *MET* alterations is not very efficient, especially for *MET*ex14 skipping mutation. The main reason is that DNA sequencing can only detect the genomic sequence alterations to predict or postulate a possible splicing result, which will not confirm the actual *MET*ex14 skipping event.

There are two DNA-based NGS technologies commonly used for genetic diagnosis of tumors currently. The first method is amplicon-based methods, which uses primers to capture the sequences of target genes in genomic regions by multiplex polymerase chain reaction (PCR) amplification (22). This method has a relatively shorter detection time and can better capture some regions that are difficult to sequence, but it is prone to sequencing distortion for regions with small insertion/deletions (indels), homopolymers, and allele loss. Amplicon-based method may fail to identify all *MET*ex14 skipping mutation, mainly because amplification primers are not designed for high-quality sequencing and fail to capture key mutation sites. In the case of single-nucleotide variant or small indels in the primer region, the primers may not bind due to mismatches, eventually leading to allele dropout (20). In addition, the binding site of the primer may also be lost if there is an entire deletion in the genomic region. In other words, the location and size of the genetic alterations that cause the *MET*ex14 skipping mutation may lead to allelic dropout and false-negative results, which ultimately lead to a low detection rate of *MET*ex14 skipping mutation using the Amplicon-based method (23). Another approach is hybrid capture-based method, which uses long biotinylated oligonucleotides to hybridize target regions in the genome and enable flanking regions to be sequenced (22). These probes are significantly longer than the PCR primers used in the amplicon-based method and thus can tolerate binding site mismatches without interfering with target hybridization, which can avoid the problem of allelic deletions seen in amplicon based methods (22). Chen et al. (24) reported that hybrid capture-based method is the preferred method to avoid common allele deletions caused by amplicon-based assays.

Unlike the detection mechanism of DNA NGS, RNA NGS can detect fusion of *MET*ex13 to 15, which is a common consequence of any altered splicing mechanism or deletion (13). Thus, it has the advantage of theoretically detecting all genomic events that lead to *MET*ex14 skipping mutation (15). Subramanian et al. (22) and Socinski et al. (21) reported that the accuracy and detection rate of RNA NGS were higher than that of DNA NGS in the detection of *MET*14 mutations. Davies et al. (15) found that the detection rate of RNA NGS was 4.2% (17/404) for *MET*ex14 skipping mutation, which was significantly higher than DNA NGS (1.3%, 11/856). But there are also problems with RNA NGS. RNA is less stable than DNA, which limits the shelf life of the tissue. In addition, interpretation of RNA NGS results poses challenges due to the high variability of mRNA expression between nonmalignant and tumor tissues (25). Teishikata et al. (26) also reported that the differences of *MET*ex14 skipping mutation detection not only exist between different sequencing technologies, but also between different NGS platforms. In short, the detection of *MET*ex14 skipping mutation by DNA NGS and RNA NGS has its own advantages and disadvantages, and it is possible to obtain more comprehensive and accurate information about *MET*ex14 skipping mutation by using dual-omics detection for some lung sarcomatoid carcinomas.

As mentioned above, the incidence of *MET*ex14 skipping mutation ranged from 22% to 31.8% in PSC, a higher mutation rate than in other types of NSCLC (16, 27). Li et al. (28) reported that in a study of 77 PSC patients, patients with advanced PSC and a positive *MET*ex14 skipping mutation had a faster rate of disease progression than patients without any driver gene mutation, and they have a median PFS of not yet reached *vs*. 3.97 months during follow-up (P =0.017). Therefore, the efficacy of molecular targeted therapy for *MET* is a hot topic at present, and many clinical studies of drugs are being carried out. Crizotinib, a multi-target TKI covering *MET*, has been reported in some small retrospective studies to treat advanced PSC with *MET*ex14 mutation. Unfortunately, these data have not been analyzed independently from other non-small cells (5). Capmatinib is an oral, potent, and selective *MET* inhibitor that has been approved by the FDA for the treatment of NSCLC patients with the *MET*ex14 mutation. A phase I single-arm trial (NCT01324479) enrolled four PSC patients with *MET*ex14 mutations, including one with stable disease and three with partial response (29). Tepotinib, the first highly selective TKI for *MET*ex14 mutation approved for marketing in the world, enrolled 2 patients with PSC out of 152 patients in the VISION study (open-label, phase 2), but the trial data were not analyzed by pathological type (30). Savolitinib is a highly selective *MET* TKI, which is the first and currently the only approved selective *MET* inhibitor in China. A multicenter, single-arm, open-label, phase 2 study of savolitinib enrolled a total of 70 patients with NSCLC who had *MET*ex14 skipping mutation (25 of whom were patients with PSC). The mPFS of PSC subgroup was 5.5 months, mOS was 10.6 months, ORR was 50%, and disease control rate (DCR) was 90% (31). It is the only *MET* inhibitor with PSC population data, which has brought a breakthrough for the treatment of PSC patients.

## *MET* amplification

*MET* amplification is also known as *MET* gene replication, mainly acquired by chromosomal 7 or local regions duplication. Aberrant chromosomal 7 replication, containing *MET* gene, always results in segmental chromosomal polysomy. The presence of polysomy can lead to the contiguous gene amplification, including *MET* and other genes on the affected chromosomal region. However, local amplification is usually caused by regionally cryptic copy number gain, but not microscopic chromosomal duplication.

Now there is no unified diagnostic criterion for *MET* amplification, with different cut-offs for different detection methods, such as immunohistochemistry (IHC), fluorescence *in situ* hybridisation (FISH), quantitative Real-Time reverse transcriptase-PCR (qRT-PCR), or NGS (Next Generation Sequencing). However, IHC appears to be a poor screen for *MET* amplification since MET IHC-positive cases may be *MET* amplification negative and vice versa (32). qRT-PCR has been used to detect *MET* amplification, although it isn’t well characterized compared to FISH and NGS (33).

Traditionally, *MET* amplification was detected by FISH with *MET* gene copy number (GCN) or the ratio of *MET* to chromosome enumerating probe against chromosome 7 (CEP7). Generally, *MET* GCN≥5 or *MET :* CEP7≥2.0 is used as the FISH criteria for *MET* amplification (3, 11). *MET :* CEP7 is more accurate than GCN, for *MET :* CEP7 is able to distinguish the true *MET* amplifications and *MET* polysomy. The degree of amplification were categorized into three groups by *MET :* CEP7, low (≥1.8 to ≤2.2), intermediate (>2.2 to <5) and high (≥5) or low (≥1.8 to ≤2.2), intermediate (>2.2 to <4) and high (≥4) (34, 35). In general, the *MET :* CEP7 ≥5 was identified as an appropriate cut-off with no overlap with other oncogenes compared with low and intermediate groups and seems to be the strongest predictor of *MET*-driven tumors (13, 36). Additionally, the targeted therapies, like crizotinib, showed more effective in patients with high *MET* amplification than low and intermediate categories (35). Now, NGS is widely used in detecting *MET* amplification. *MET* amplification was commonly defined by copy number fold change of 1.8x or more by NGS. Similar to FISH, the cut-offs may vary significantly between different assays. The main limitation of NGS is that the result is highly dependent on the quality of the sample and, more importantly, the amount of non-tumor DNA from the non-tumor cells in the sample (37).

*De novo MET* amplification (primary *MET* amplification) occurs in about 1-5% of non-small-cell lung cancers (NSCLC), and acquired *MET* amplification (secondary *MET* amplification) are typically identified in about 5-20% patients with oncogene-positive NSCLC following resistance to tyrosine kinase inhibitors (TKIs), such as EGFR TKIs (38, 39). Until now, there was no large-scale prospective or retrospective studies of PSC with *MET* amplification. The frequency of *MET* amplification in PSC has rarely been reported. Mignard et al. (40) reported *MET* amplification in about 8.5% of PSC patients, Tong et al. (41) about 13.6%, and Liu et al. (42) about 4.8%. These variations may depend on the sample size and different methods used in each study.

*MET* amplification is a type of confirmed mechanisms of acquired resistance to EGFR-TKIs and ALK inhibitors in NSCLC (43). Several case reports of *MET* amplification with PSC have been published. Wang et al. reported a 74-year-old female PSC patient with co-existing mutation in exon 21 L858R of *EGFR* and *MET* amplification at diagnosis (44). Combination of EGFR and MET inhibitors, gefitinib and crizotinib, respectively, were used. The patient acquired a partial response and remained stable for 9.7 months after terminated treatment. This observation highlights the importance of genetic testing and paves the way for combined targeting strategies in PSC. This was the first reported case of PSC patient with concurrent *EGFR* mutation and *MET* amplification prior to treatment, who may benefit from combination EGFR and MET inhibitors.

He et al. (44) Reported that a 62-year-old male patient carrying two rare *EGFR* mutations, exon 18 L719S and exon 19 L797S. After application of afatinib for 6 months, the patient experience disease progression. NGS found new acquired *MET* amplification with original mutations of EGFR exon 18 L719S and exon 19 L797S. Then the patient was treated with afatinib combined with crizotinib, with a result of a partial response of the disease. Combined therapies may be efficient for the *MET* amplification PSC patients with concurrent mutation of other oncogenes or secondary to resistance of previous treatment of TKIs. More large-scale clinical trials are required to confirm the findings.

## *MET* overexpression

*MET* ovexpression is thought to be one of the earliest *MET* dysregulation event in oncogenic process. *MET* can be found transcriptionally overexpressed in the presence of hypoxia and inflammation, thereby activating proliferation, reducing apoptosis, promoting migration, these all contributing to tumorigenesis (45). *MET* overexpression is present in many types of cancers, such as epithelial, mesenchymal and hematological malignancies. In addition, *MET* can be overexpressed in cancers with activated genomic signature, including those with primary and/or secondary *MET* amplifications or *MET*ex14 skipping mutation. Until now, *MET* TKIs showed little effect on patients with *MET* overexpression partially due to discrepancies in its causes (1). Fortunately, new therapeutic targets for *MET* are being explored, including biparatopic antibodies (targeting two different epitopes on the same target protein), antibodies and ADCs combinations.

The most commonly used detection method of *MET* expression is IHC with kinds of antibodies, consisted of monoclonal antibodies, polyclonal antibodies and antibodies to phosphorylated MET (46). Generally, the IHC staining of MET were always assessed by the pathologists, providing the basis for various semiquantitative scoring systems of MET protein expression and overexpression. The degree of MET expression is usually quantified as a staining score from 0 to 3+ (47). IHC 1+ indicates MET expression, while *MET* overexpression is defined as IHC 2+ and 3+ (47). The H-score is another typical scoring system calculated by multiplying the percentages of cells of MET expression with their staining intensity score, and ranging from 0 to 300 (43). The score ≥200 usually indicates *MET* overexpression, but different thresholds vary between different clinical studies (43).

*MET* overexpression has been reported with high frequencies in NSCLC, ranging from 22.2-74.5%, and seems to portend poorer prognosis (4). In contrast, studies on *MET* overexpression in PSCs are limited. Liu et al. (42) found *MET* overexpression in 20.2% PSCs and carried survival analysis of *MET* gene alterations and protein expression in Chinese PSCs with only surgery but no MET TKI treatment. They found that *MET* amplification suffered shorter mOS, while *MET*ex14 skipping mutation and overexpression didn’t affect patients’ survival. The studies showed different prognostic value of *MET* overexpression between PSCs and NSCLCs.

Xavier Mignard et al. (40) found *MET* overexpression as a poor predictor of *MET* amplifications or exon 14 mutations in PSCs. They reported that *MET* exon 14 mutations could cause loss of ubiquitination and improve the presence of c-MET membrane, while lack of association between them and *MET* overexpression remains to be understood. These results may be induced by the different genomic backgrounds between PSCs and NSCLCs, or other mechanisms in the oncogenesis of *MET* exon 14 mutations. The lack of clinical trials about PSCs results in the limited understanding of the biological mechanisms, development, diagnosis, prognosis and treatment of the tumors (48). We are looking forward to more relevant clinical studies.

## Conclusion

PSC is a subtype of NSCLC with unique malignant biological behavior. The symptoms are not specific, and PSC is often found in the advanced stage. It is not sensitive to traditional chemotherapy and radiotherapy, and the expected survival time is short, and the prognosis is poor. It is most likely that the treatment of PSC should continue to follow the treatment guidelines for advanced NSCLC. Molecular pathology tests, including IHC and NGS, are valuable in determining the prognosis of patients with PSC and helping to determine the treatment of NSCLC patients. *MET* alterations occur more frequently in PSC than in other types of NSCLC, and *MET*ex14 skipping mutation is the most common type. The existing clinical data have preliminarily confirmed the efficacy of *MET*-TKI in PSC patients with *MET* alteration, among which the clinical study of Savolitinib has enrolled the largest proportion of PSC patients and achieved relatively good efficacy, but more clinical research is still needed. The multi-disciplinary team may maximize the optimal treatment options for patients with advanced PSC.

## Author contributions

CG wrote the section of introduction and *MET* mutation. HX and JZhang designed the review and revised the manuscript. KQ wrote the section of wild-type *MET*. JW wrote the section of *MET* amplification. JZhao wrote the section of *MET* overexpression. YC wrote the section of conclusion. All authors contributed to the article and approved the submitted version.

## Conflict of interest

The authors declare that the research was conducted in the absence of any commercial or financial relationships that could be construed as a potential conflict of interest.

## Publisher’s note

All claims expressed in this article are solely those of the authors and do not necessarily represent those of their affiliated organizations, or those of the publisher, the editors and the reviewers. Any product that may be evaluated in this article, or claim that may be made by its manufacturer, is not guaranteed or endorsed by the publisher.
